# Supplementary material for: Prediction of odds for emergency cesarean section: A secondary analysis of the CHILD term birth cohort study
Source: PLoS One. 2022 Oct 6;17(10):e0268229. doi: 10.1371/journal.pone.0268229 (PMC9536615; doi:10.1371/journal.pone.0268229)
Supplement: S1 File — (DOCX) [file pone.0268229.s001.docx]

**S1 Table.** Demographic, antenatal and obstetric characteristics of Training and Validation data set

| Characteristics | Training  (n=2,150) | Validation  (n=530) |
| --- | --- | --- |
| Emergency CS (n, %) | 283 (80%) | 82 (20%) |
| Maternal Age (years) (mean ± SD) | 32.14 ± 4.68 | 31.84 ± 4.60 |
| Maternal Height (cm) (mean ± SD) | 165.29 ± 6.92 | 165.59 ± 6.76 |
| Maternal Weight (kg) (mean ± SD) | 69.24 ± 16.72 | 68.24 ± 16.67 |
| BMI in kg/m^2^ (mean ± SD) | 25.32 ± 5.81 | 25.17 ± 5.85 |
| Hospitals CS rate CHILD cohort (mean ± SD) | 5.94 ± 3.20 | 6.12 ± 3.17 |
| Increased CESD-score (Ref: <10) | 608 (28%) | 134 (25%) |
| Gestational Age (weeks) |  |  |
| 37 | 132 (6%) | 30 (6%) |
| 38 | 248 (12%) | 47 (9%) |
| 39 | 480 (22%) | 136 (26%) |
| 40 | 687 (32%) | 159 (30%) |
| 41 | 492 (24%) | 126 (24%) |
| ≥42 | 99 (4%) | 25 (5%) |
| Gravida |  |  |
| G1 | 840 (39%) | 221 (41%) |
| G2 | 676 (32%) | 162 (31%) |
| G3 | 341 (16%) | 83 (16%) |
| G4 | 161 (7%) | 36 (7%) |
| ≥G5 | 131 (6%) | 27 (5%) |
| Maternal Ethnicity |  |  |
| Caucasian | 1197 (73%) | 294 (73%) |
| Others | 433 (27%) | 106 (27%) |
| Marital status |  |  |
| Married or Common Law | 1929 (85%) | 491 (87%) |
| Single (Never been married) | 112 (5%) | 25 (4%) |
| Divorced/Widowed/ Separated | 109 (10%) | 14 (9%) |
| Socioeconomic status |  |  |
| <$60,000 | 382 (20%) | 98 (21%) |
| ≥ $60,000 | 1483 (80%) | 367 (79%) |
| Maternal Education |  |  |
| No education beyond high school | 197 (10%) | 42 (8%) |
| Some post secondary/ college | 427 (21%) | 108 (21%) |
| University degree | 1,433 (69%) | 361 (71%) |
| Maternal smoking history |  |  |
| Yes | 68 (4%) | 16 (4%) |
| Hypertensive Disorders of Pregnancy |  |  |
| Yes | 81 (4%) | 14 (3%) |
| Gestation Diabetes |  |  |
| Yes | 97 (5%) | 24 (5%) |
| Previous vaginal delivery |  |  |
| First Born | 1,157 (54%) | 302 (57%) |
| Subsequent Born | 993 (46%) | 226 (43%) |
| Child Sex |  |  |
| Male | 1,130 (53%) | 285 (54%) |
| Female | 1,017 (47%) | 245 (46%) |

*=Vaginal delivery was used as a reference and compared with emergency CS and scheduled CS**.**

**p-values <0.05 in bold**; SD=standard deviation; BMI=body mass index

**S2 Table**. Reference studies reporting the risk of caesarean section in nulliparous and multiparous

|  | **Obstetric** | **Non-obstetric** |
| --- | --- | --- |
| **Antepartum** | Maternal factors  -gestational hypertension [1–3]  -preeclampsia, eclampsia [2,3]  -gestational diabetes [2–4]  -premature rupture of membrane [5]  -parity [3,6]  -cephalo pelvis disproportion [3,7]  -previous vaginal delivery [3,7]  -in-vitro fertilization (IVF) [2,8]  Others  -gestation age [3] | Maternal factors  -age [6,7,9–13]  -height [3,7,9,10,14]  -pre-pregnancy weight/ BMI and pregnancy weight gain [2,3,7,10,11,15–17]  -socio economic status [6]  -race/ethnicity [3,7]  -education [6,18]  -smoking [2]  -maternal stress, anxiety, depression score [10,19]  -chronic hypertension [3,20]  -diabetes [3,4]  -sleep disorder (obstructive sleep apnoea) [21]  -maternal request [22]  Fetal factors  -macrosomia [3,10,23]  -position (occiput posterior) [3,10]  -presentation (breech) [3,10]  -gender [24]  Others  -exercise, diet [25,26]  -vitamin D supplementation [27,28]  -senior healthcare professionals/ healthcare institution [3,29]  -healthcare insurance [30] |
| **Intrapartum** | Maternal factors  -cervical dilatation (Bishop score) [3,10,18]  Others  -epidural analgesia [10,31,32]  -induction of labour (IOL)  [3,10,33–36]  -augmentation of labour [10]  -dystocia or failure to progress [3,10]  -fetal distress [3,18]  -acupressure [37] | Others  -Cardiotocography monitoring [38] |

**S3 Table.** Multiple logistic regression results of CS include demographic, antenatal physical and obstetric characteristics in nulliparous and multiparous cohort

|  | Nulliparous (n=1099) | | Multiparous (n=1737) | |
| --- | --- | --- | --- | --- |
|  | Odds Ratio | 95% CI | Odds Ratio | 95% CI |
| Centered Age (years) | **1.06** | **1.03-1.10** | **1.06** | **1.03-1.10** |
| Centered Height (cm) | **0.94** | **0.92-0.96** | **0.96** | **0.94-0.98** |
| BMI in kg/m^2^ | **1.05** | **1.02-1.07** | **1.05** | **1.03-1.08** |
| CESD-score (ref: <10) | **1.44** | **1.01-2.05** | **1.46** | **1.08-1.98** |
| Hospital CS rate (CHILD) | **1.09** | **1.05-1.14** | **1.06** | **1.03-1.09** |
| Hypertensive Disorders of Pregnancy | **1.93** | **1.02-3.67** | 1.09 | 0.55-2.21 |
| Previous vaginal delivery | **-** | **-** | **0.46** | **0.34-0.62** |
| AUC | 0.69 | 0.65-0.73 | 0.70 | 0.66-0.73 |

**P-values <0.05 in bold;** AUC= area under curve; OR= odds ratio; CI= confidence interval;

**S4 Table.** Multiple logistic regression results include demographic, antenatal physical and obstetric characteristics in overall cohort independent of the parity: (Training and validation dataset) excluded CESD-score

|  |  | Emergency CS  (Training, n=2150) | | | | Emergency CS  (Validation, n=530) | | | | | |
| --- | --- | --- | --- | --- | --- | --- | --- | --- | --- | --- | --- |
|  | Coefficient | | | Odds Ratio | 95% CI | | Coefficient | | Odds Ratio | 95% CI |  |
| Centered Age (years) | | | **0.05** | **1.05** | **1.02-1.08** | | **0.13** | | **1.14** | **1.07-1.22** |  |
| Centered Height (cm) | | | **-0.06** | **0.94** | **0.92-0.96** | | **-0.06** | | **0.94** | **0.91-0.98** |  |
| BMI in kg/m^2^ | | | **0.04** | **1.04** | **1.02-1.07** | | **0.08** | | **1.08** | **1.04-1.13** |  |
| CESD-score (ref: <10) | | | **-** | **-** | **-** | | **-** | | **-** | **-** |  |
| Hospital CS rate (CHILD) | | | 0.04 | 1.04 | 0.99-1.09 | | **0.16** | | **1.17** | **1.07-1.29** |  |
| Hypertensive Disorders of Pregnancy | | | **0.56** | **1.76** | **1.00-3.09** | | 0.16 | | 1.17 | 0.32-4.27 |  |
| Previous vaginal delivery | | | **-1.56** | **0.21** | **0.15-0.30** | | **-1.63** | | **0.20** | **0.10-0.38** |  |
| Constant | | | **-2.78** |  |  | | **-4.39** | |  |  |  |
| AUC | | |  | 0.74 | 0.71-0.77 | |  | | 0.78 | 0.73-0.83 |  |
| Sensitivity | | |  | 12% |  | | |  | 12% |  |  |
| Specificity | | |  | 99% |  | | |  | 99% |  |  |
| Positive Predictive Value | | |  | 38% |  | | |  | 69% |  |  |
| Negative Predictive Value | | |  | 87% |  | | |  | 86% |  |  |
| Accuracy | | |  | 87% |  | | |  | 85% |  |  |

**P-values <0.05 in bold;** AUC= area under curve; OR= odds ratio; CI= confidence interval;

**S1 Figure.** Flow diagram of the statistical analysis

Descriptive data analysis
(mean ± standard deviation (SD) or median ± interquartile range (IQR)

Non-stratified cohort

Multiparous cohort

Logistic Regression model

Modified Antenatal Scoring System

Logistic Regression Model

Nulliparous cohort

Logistic Regression Model

**S1 Appendix:** PubMed July 2020

| Search | Query | Results |
| --- | --- | --- |
| #7 | #1 AND #2 AND #3 AND #5 | 1843 |
| #6 | #1 AND #2 AND #4 AND #5 | 356 |
| #5 | ((systematic review) OR meta analysis) AND metaanalysis | 125491 |
| #4 | (((((((nulliparous) OR (first time pregnant)) OR (first time mother)) OR (first time deliver)) OR (nonparturitive)) OR (first pregnancy)) OR (nonparturient)) OR (nonparous) | 194232 |
| #3 | (((((((((vaginal birth after cesarean) OR (vbac)) OR (vaginal birth)) OR (vaginal deliver)) OR (trial of labor)) OR (postcesarean)) OR (postcaesarean)) OR (c section)) OR (abdominal deliver)) OR (uterine scar) | 82725 |
| #2 | (((((((Cesarean Section) OR (cesarean)) OR (caesarean abdominal deliver)) OR (caesarea)) OR (cesarea)) OR (c section)) OR (CS)) OR (abdominal deliver) | 519462 |
| #1 | (((((((((risk factors) OR (predict)) OR (model)) OR (predictors)) OR (indicators)) OR (determinants)) OR (risk score)) OR (risk management)) OR (factor)) OR (risk) | 16614138 |

**References**

1. Patel RR, Peters TJ, Murphy DJ, the ALSPAC ST. Prenatal risk factors for Caesarean section. Analyses of the ALSPAC cohort of 12 944 women in England. Int J Epidemiol [Internet]. 2005;34(2):353–67. Available from: https://doi.org/10.1093/ije/dyh401

2. Salahuddin M, Mandell DJ, Lakey DL, Eppes CS, Patel DA. Maternal risk factor index and cesarean delivery among women with nulliparous, term, singleton, vertex deliveries, Texas, 2015. Birth. 2019 Mar;46(1):182–92.

3. Wu Y, Kataria Y, Wang Z, Ming WK, Ellervik C. Factors associated with successful vaginal birth after a cesarean section: a systematic review and meta-analysis. BMC Pregnancy Childbirth. 2019 Oct 17;19(1):360-019-2517-y.

4. Yu L, Zeng XL, Cheng ML, Yang GZ, Wang B, Xiao ZW, et al. Quantitative assessment of the effect of pre-gestational diabetes and risk of adverse maternal, perinatal and neonatal outcomes. Oncotarget. 2017 May 11;8(37):61048–56.

5. Bond DM, Middleton P, Levett KM, van der Ham DP, Crowther CA, Buchanan SL, et al. Planned early birth versus expectant management for women with preterm prelabour rupture of membranes prior to 37 weeks’ gestation for improving pregnancy outcome. Cochrane database Syst Rev. 2017 Mar 3;3(3):CD004735.

6. Jahnke JR, Houck KM, Bentley ME, Thompson AL. Rising rates of cesarean delivery in Ecuador: Socioeconomic and institutional determinants over two decades. Birth. 2019 Jun;46(2):335–43.

7. Grobman WA, Lai Y, Landon MB, Spong CY, Leveno KJ, Rouse DJ, et al. Can a prediction model for vaginal birth after cesarean also predict the probability of morbidity related to a trial of labor? Am J Obstet Gynecol. 2009 Jan;200(1):56.e1-56.e6.

8. Moreno-Sepulveda J, Checa MA. Risk of adverse perinatal outcomes after oocyte donation: a systematic review and meta-analysis. J Assist Reprod Genet. 2019 Oct;36(10):2017–37.

9. Grobman WA, Lai Y, Landon MB, Spong CY, Leveno KJ, Rouse DJ, et al. Development of a nomogram for prediction of vaginal birth after cesarean delivery. Obstet Gynecol. 2007 Apr;109(4):806–12.

10. Lowe NK. A review of factors associated with dystocia and cesarean section in nulliparous women. J Midwifery Womens Health. 2007;52(3):216–28.

11. Naftalin J, Paterson-Brown S. A pilot study exploring the impact of maternal age and raised body mass index on caesarean section rates. J Obstet Gynaecol [Internet]. 2008;28(4):394–7. Available from: http://login.ezproxy.library.ualberta.ca/login?url=http://search.ebscohost.com/login.aspx?direct=true&db=a9h&AN=32965055&site=eds-live&scope=site

12. Pinheiro RL, Areia AL, Mota Pinto A, Donato H. Advanced Maternal Age: Adverse Outcomes of Pregnancy, A Meta-Analysis. Acta Med Port. 2019 Mar 29;32(3):219–26.

13. Smith GC, Dellens M, White IR, Pell JP. Combined logistic and Bayesian modeling of cesarean section risk. Am J Obstet Gynecol. 2004 Dec;191(6):2029–34.

14. Prasad M, Al-Taher H. Maternal height and labour outcome. J Obstet Gynaecol. 2002 Sep;22(5):513–5.

15. Oteng-Ntim E, Mononen S, Sawicki O, Seed PT, Bick D, Poston L. Interpregnancy weight change and adverse pregnancy outcomes: a systematic review and meta-analysis. BMJ Open. 2018 Jun 4;8(6):e018778-2017–018778.

16. Poobalan AS, Aucott LS, Gurung T, Smith WC, Bhattacharya S. Obesity as an independent risk factor for elective and emergency caesarean delivery in nulliparous women--systematic review and meta-analysis of cohort studies. Obes Rev. 2009 Jan;10(1):28–35.

17. Timmermans YEG, van de Kant KDG, Oosterman EO, Spaanderman MEA, Villamor-Martinez E, Kleijnen J, et al. The impact of interpregnancy weight change on perinatal outcomes in women and their children: A systematic review and meta-analysis. Obes Rev. 2020 Mar;21(3):e12974.

18. Karabulut A, Derbent AU, Yildirim M, Simavli S, Turhan NÖ. Evaluation of risk factors and effect of physical activity in caesarean section in nulliparous women. J Matern Fetal Neonatal Med. 2012 Aug;25(8):1456–9.

19. Sydsjö G, Möller L, Lilliecreutz C, Bladh M, Andolf E, Josefsson A. Psychiatric illness in women requesting caesarean section. BJOG. 2015 Feb;122(3):351–8.

20. Bramham K, Parnell B, Nelson-Piercy C, Seed PT, Poston L, Chappell LC. Chronic hypertension and pregnancy outcomes: systematic review and meta-analysis. BMJ. 2014 Apr 15;348:g2301.

21. Brown NT, Turner JM, Kumar S. The intrapartum and perinatal risks of sleep-disordered breathing in pregnancy: a systematic review and metaanalysis. Am J Obstet Gynecol. 2018 Aug;219(2):147-161.e1.

22. O’Donovan C, O’Donovan J. Why do women request an elective cesarean delivery for non-medical reasons? A systematic review of the qualitative literature. Birth. 2018 Jun;45(2):109–19.

23. Boulvain M, Irion O, Dowswell T, Thornton JG. Induction of labour at or near term for suspected fetal macrosomia. Cochrane database Syst Rev. 2016 May 22;2016(5):CD000938.

24. Guan P, Tang F, Sun G, Ren W. Prediction of emergency cesarean section by measurable maternal and fetal characteristics. J Investig Med. 2020 Mar;68(3):799–806.

25. Chen I, Opiyo N, Tavender E, Mortazhejri S, Rader T, Petkovic J, et al. Non-clinical interventions for reducing unnecessary caesarean section. Cochrane database Syst Rev. 2018 Sep 28;9(9):CD005528.

26. Shepherd E, Gomersall JC, Tieu J, Han S, Crowther CA, Middleton P. Combined diet and exercise interventions for preventing gestational diabetes mellitus. Cochrane database Syst Rev. 2017 Nov 13;11(11):CD010443.

27. Pérez-López FR, Pasupuleti V, Mezones-Holguin E, Benites-Zapata VA, Thota P, Deshpande A, et al. Effect of vitamin D supplementation during pregnancy on maternal and neonatal outcomes: a systematic review and meta-analysis of randomized controlled trials. Fertil Steril. 2015 May;103(5):1278-88.e4.

28. van der Pligt P, Willcox J, Szymlek-Gay EA, Murray E, Worsley A, Daly RM. Associations of Maternal Vitamin D Deficiency with Pregnancy and Neonatal Complications in Developing Countries: A Systematic Review. Nutrients. 2018 May 18;10(5):640. doi: 10.3390/nu10050640.

29. Reid HE, Hayes D, Wittkowski A, Vause S, Whitcombe J, Heazell A. The effect of senior obstetric presence on maternal and neonatal outcomes in UK NHS maternity units: a systematic review and meta-analysis. BJOG. 2017 Aug;124(9):1321–30.

30. Hoxha I, Syrogiannouli L, Braha M, Goodman DC, da Costa BR, Jüni P. Caesarean sections and private insurance: systematic review and meta-analysis. BMJ Open. 2017 Aug 21;7(8):e016600-2017–016600.

31. Anim-Somuah M, Smyth RM, Cyna AM, Cuthbert A. Epidural versus non-epidural or no analgesia for pain management in labour. Cochrane database Syst Rev. 2018 May 21;5(5):CD000331.

32. Sng BL, Leong WL, Zeng Y, Siddiqui FJ, Assam PN, Lim Y, et al. Early versus late initiation of epidural analgesia for labour. Cochrane database Syst Rev. 2014 Oct 9;(10):CD007(10):CD007238.

33. Grobman WA, Rice MM, Reddy UM, Tita ATN, Silver RM, Mallett G, et al. Labor Induction versus Expectant Management in Low-Risk Nulliparous Women. N Engl J Med. 2018 Aug 9;379(6):513–23.

34. Middleton P, Shepherd E, Crowther CA. Induction of labour for improving birth outcomes for women at or beyond term. Cochrane database Syst Rev. 2018 May 9;5:CD004945.

35. Saccone G, Della Corte L, Maruotti GM, Quist-Nelson J, Raffone A, De Vivo V, et al. Induction of labor at full-term in pregnant women with uncomplicated singleton pregnancy: A systematic review and meta-analysis of randomized trials. Acta Obstet Gynecol Scand. 2019 Aug;98(8):958–66.

36. Walker KF, Bugg G, Macpherson M, McCormick C, Wildsmith C, Smith G, et al. Induction of labour versus expectant management for nulliparous women over 35 years of age: a multi-centre prospective, randomised controlled trial. BMC Pregnancy Childbirth. 2012 Dec 11;12:145.

37. Makvandi S, Mirzaiinajmabadi K, Sadeghi R, Mahdavian M, Karimi L. Meta-analysis of the effect of acupressure on duration of labor and mode of delivery. Int J Gynaecol Obstet Off organ Int Fed Gynaecol Obstet. 2016 Oct;135(1):5–10.

38. Alfirevic Z, Devane D, Gyte GM, Cuthbert A. Continuous cardiotocography (CTG) as a form of electronic fetal monitoring (EFM) for fetal assessment during labour. Cochrane database Syst Rev. 2017 Feb 3;2(2):CD006066.
