# Supplementary material for: Prediction of odds for emergency cesarean section: A secondary analysis of the CHILD term birth cohort study
Source: PLoS One. 2022 Oct 6;17(10):e0268229. doi: 10.1371/journal.pone.0268229 (PMC9536615; doi:10.1371/journal.pone.0268229)
Supplement: Attachment — Submitted filename: 2021-08-15 PLOS One R1 Response To Reviewers.docx [file pone.0268229.s002.docx]

PONE-D-21-10805
Prediction of odds for emergency cesarean section: a secondary analysis of the CHILD term birth cohort study
PLOS ONE

Dear Dr. Mandhane,

Thank you for submitting your manuscript to PLOS ONE. After careful consideration, we feel that it has merit but does not fully meet PLOS ONE’s publication criteria as it currently stands. Therefore, we invite you to submit a revised version of the manuscript that addresses the points raised during the review process.

Please submit your revised manuscript by Aug 15 2021 11:59PM. If you will need more time than this to complete your revisions, please reply to this message or contact the journal office at [plosone@plos.org](mailto:plosone@plos.org). When you're ready to submit your revision, log on to <https://www.editorialmanager.com/pone/> and select the 'Submissions Needing Revision' folder to locate your manuscript file.

Please include the following items when submitting your revised manuscript:

- A rebuttal letter that responds to each point raised by the academic editor and reviewer(s). You should upload this letter as a separate file labeled 'Response to Reviewers'.
- A marked-up copy of your manuscript that highlights changes made to the original version. You should upload this as a separate file labeled 'Revised Manuscript with Track Changes'.
- An unmarked version of your revised paper without tracked changes. You should upload this as a separate file labeled 'Manuscript'.

If you would like to make changes to your financial disclosure, please include your updated statement in your cover letter. Guidelines for resubmitting your figure files are available below the reviewer comments at the end of this letter.

If applicable, we recommend that you deposit your laboratory protocols in [protocols.io](http://protocols.io/) to enhance the reproducibility of your results. Protocols.io assigns your protocol its own identifier (DOI) so that it can be cited independently in the future. For instructions see: <http://journals.plos.org/plosone/s/submission-guidelines#loc-laboratory-protocols>. Additionally, PLOS ONE offers an option for publishing peer-reviewed Lab Protocol articles, which describe protocols hosted on [protocols.io](http://protocols.io/). Read more information on sharing protocols at <https://plos.org/protocols?utm_medium=editorial-email&utm_source=authorletters&utm_campaign=protocols>.

We look forward to receiving your revised manuscript.

Kind regards,

Eduardo Ortiz-Panozo, MD; MSc
Academic Editor
PLOS ONE

Journal Requirements:

When submitting your revision, we need you to address these additional requirements.

1. Please ensure that your manuscript meets PLOS ONE's style requirements, including those for file naming. The PLOS ONE style templates can be found at 
<https://journals.plos.org/plosone/s/file?id=wjVg/PLOSOne_formatting_sample_main_body.pdf> and 
<https://journals.plos.org/plosone/s/file?id=ba62/PLOSOne_formatting_sample_title_authors_affiliations.pdf>

2. Thank you for including your ethics statement: "Ethics approval was obtained from local authorized review board of each CHILD study center and McMaster University. A separate ethics approval was obtained for this secondary data analysis (Pro00092920)."

a) Please amend your current ethics statement to include the full name of the ethics committee/institutional review board(s) that approved your specific study.

Response: Added in Line 119-123

"Ethics approval was obtained from the research ethics board of each CHILD study center (University of Alberta research ethics board, McMaster University research ethics board, Hospital for Sick Children research ethics board, University of Manitoba research ethics board, and University of British Columbia research ethics board) in addition to the McMaster University research ethics board. ”

c) Please provide additional details regarding participant consent. In the ethics statement in the Methods and online submission information, please ensure that you have specified (1) whether consent was informed and (2) what type you obtained (for instance, written or verbal, and if verbal, how it was documented and witnessed). If your study included minors, state whether you obtained consent from parents or guardians. If the need for consent was waived by the ethics committee, please include this information.

Response: Line 117-118

“Pregnant women provided written informed consent to participate in the CHILD study”

If you are reporting a retrospective study of medical records or archived samples, please ensure that you have discussed whether all data were fully anonymized before you accessed them and/or whether the IRB or ethics committee waived the requirement for informed consent. If patients provided informed written consent to have data from their medical records used in research, please include this information.

Response: Line 125-129

“Patients provided informed written consent to have data from their medical records used for the CHILD study. A separate ethics approval was obtained from the University of Alberta Research Ethics Board for this secondary data analysis (Pro00092920). The data used for this study was de-identified (no participant were identifiers included in the dataset) prior to being released for analysis.”

Once you have amended this/these statement(s) in the Methods section of the manuscript, please add the same text to the “Ethics Statement” field of the submission form (via “Edit Submission”).

For additional information about PLOS ONE ethical requirements for human subjects research, please refer to <http://journals.plos.org/plosone/s/submission-guidelines#loc-human-subjects-research>.

Response: We have provided this information in the responses and will update the manuscript upon resubmission.

3. In ethics statement in the manuscript and in the online submission form, please provide additional information about the patient records/samples used in your study. Specifically, please ensure that you have discussed whether all data/samples were fully anonymized before you accessed them and/or whether the IRB or ethics committee waived the requirement for informed consent. If patients provided informed written consent to have data/samples from their medical records used in research, please include this information.

Response: We have clarified the ethics information for this project in the manuscript and in the ethics statement field of the submission.

"Ethics approval was obtained from the research ethics board of each CHILD study center (University of Alberta research ethics board, McMaster University research ethics board, Hospital for Sick Children research ethics board, University of Manitoba research ethics board, and University of British Columbia research ethics board) in addition to the McMaster University research ethics board. Pregnant women provided written informed consent to participate in the CHILD study. A separate ethics approval was obtained from the University of Alberta Research Ethics Board for this secondary data analysis (Pro00092920). The data used for this study was de-identified (no participant were identifiers included in the dataset) prior to being released for analysis.”

4. In your Data Availability statement, you have not specified where the minimal data set underlying the results described in your manuscript can be found. PLOS defines a study's minimal data set as the underlying data used to reach the conclusions drawn in the manuscript and any additional data required to replicate the reported study findings in their entirety. All PLOS journals require that the minimal data set be made fully available. For more information about our data policy, please see <http://journals.plos.org/plosone/s/data-availability>.

Upon re-submitting your revised manuscript, please upload your study’s minimal underlying data set as either Supporting Information files or to a stable, public repository and include the relevant URLs, DOIs, or accession numbers within your revised cover letter. For a list of acceptable repositories, please see <http://journals.plos.org/plosone/s/data-availability#loc-recommended-repositories>. Any potentially identifying patient information must be fully anonymized.

Important: If there are ethical or legal restrictions to sharing your data publicly, please explain these restrictions in detail. Please see our guidelines for more information on what we consider unacceptable restrictions to publicly sharing data: <http://journals.plos.org/plosone/s/data-availability#loc-unacceptable-data-access-restrictions>. Note that it is not acceptable for the authors to be the sole named individuals responsible for ensuring data access.

We will update your Data Availability statement to reflect the information you provide in your cover letter.

Response: The dataset used for this analysis contains human data of a potentially sensitive nature (e.g. delivery mode, anthropometrics and results from the CES-D). Data used for this analysis are available through an application to the CHILD study (<https://childstudy.ca/for-researchers/data-access/>).

5. Please include captions for your Supporting Information files at the end of your manuscript, and update any in-text citations to match accordingly. Please see our Supporting Information guidelines for more information: <http://journals.plos.org/plosone/s/supporting-information>. 

**Additional Editor Comments:**

1) Since the study aims at developing a prediction model, I suggest to include a table showing the final model's regression coefficients plus the intercept. Not as a supplementary table (i.e, Supplementary table 3) but as part of the main analysis.

Response 1: Thank you for the suggestion. We added the coefficient and the intercept values in Table 2 together with the odds ratio values.

**Table 2.** Multiple logistic regression results include demographic, antenatal physical and obstetric characteristics in overall cohort independent of the parity: (Training and validation dataset)

|  |  | Emergency CS  (Training, n=2150) | | | | Emergency CS  (Validation, n=530) | | | | | |
| --- | --- | --- | --- | --- | --- | --- | --- | --- | --- | --- | --- |
|  | Coefficient | | | Odds Ratio | 95% CI | | Coefficient | | Odds Ratio | 95% CI |  |
| Centered Age (years) | | | **0.05** | **1.06** | **1.02-1.08** | | **0.13** | | **1.14** | **1.07-1.22** |  |
| Centered Height (cm) | | | **-0.06** | **0.94** | **0.92-0.96** | | **-0.06** | | **0.94** | **0.90-0.98** |  |
| BMI in kg/m^2^ | | | **0.04** | **1.04** | **1.02-1.06** | | **0.07** | | **1.07** | **1.03-1.12** |  |
| CESD-score (ref: <10) | | | **0.37** | **1.45** | **1.07-1.96** | | **0.51** | | **1.66** | **1.01-3.15** |  |
| Hospital CS rate (CHILD) | | | 0.04 | 1.04 | 0.98-1.09 | | 0.16 | | 1.17 | 0.98-1.28 |  |
| Hypertensive Disorders of Pregnancy | | | **0.56** | **1.75** | **1.99-3.08** | | 0.28 | | 1.32 | 0.36-4.80 |  |
| Previous vaginal delivery | | | **-1.57** | **0.21** | **0.15-0.29** | | **-1.63** | | **0.20** | **0.10-0.38** |  |
| Intercept (constant) | | | -2.85 |  |  | | -4.33 | |  |  |  |
| AUC | | |  | 0.74 | 0.72-0.77 | |  | | 0.77 | 0.71-0.82 |  |
| Sensitivity | | |  | 12% |  | | |  | 13% |  |  |
| Specificity | | |  | 99% |  | | |  | 98% |  |  |
| Positive Predictive Value | | |  | 28% |  | | |  | 63% |  |  |
| Negative Predictive Value | | |  | 87% |  | | |  | 89% |  |  |
| Accuracy | | |  | 87% |  | | |  | 85% |  |  |

**P-values <0.05 in bold;** AUC= area under curve; OR= odds ratio; CI= confidence interval;

2) Please clarify how many and what variables were tested as potential predictors. What were the p-values for excluded variables?

Response 2: The additional variables tested and their p-values are presented below:

|  | p-value | |
| --- | --- | --- |
|  | Univariate | Multiple – variable |
| Enrolled sites  (Edmonton, Winnipeg, Toronto, Vancouver) | 0.22 | 0.69 |
| Maternal smoking history | 0.63 | 0.71 |
| Maternal education level | 0.47 | 0.83 |
| Maternal socioeconomic status | 0.56 | 0.22 |
| Maternal marital status | 0.21 | 0.31 |
| Maternal ethnicity | 0.02 | 0.85 |
| Maternal gestational diabetes | 0.08 | 0.54 |
| Gestational age at delivery | 0.18 | 0.58 |

3) Authors mention Table 3a and a scoring system in the text, but I could not find those results in tables.

Response 3: The scores assigned to the variables as shown in Table 3a of the manuscript and presented below. Total scores of the study population range from 0 to 14. The scores were further grouped into 4 Grades (Table 3b of the manuscript) and presented below.

Table 3a: Scoring for the Emergency CS prediction tool

| Age (years) | | Height (cm) | | BMI (kg/m2) | | CES-D score | | Living child (previous vaginal delivery) | | Pregnancy induced hypertension/ Pre-eclampsia | |
| --- | --- | --- | --- | --- | --- | --- | --- | --- | --- | --- | --- |
| Value | Score | Value | Score | Value | Score | Value | Score | Value | Score | Value | Score |
| ≤ 30 | 0 | ≤ 160 | 4 | < 18.5 | 0 | Low (<10) | 0 | Absent | 5 | Absent | 0 |
| 31-35 | 2 | 161-165 | 2 | 18.5-25 | 1 | High (≥10) | 2 | Present | 0 | Present | 2 |
| > 35 | 4 | > 165 | 0 | > 25 | 3 |  |  |  |  |  |  |

**Table 3b.** Emergency CS prediction risk scoring system

| Score | n (%) | Emergency CS (n, %) | Odds Ratio (95% CI) | Numbers Needed to Treat (NNT) |
| --- | --- | --- | --- | --- |
| Grade 0 (0-5 points) | 459 (22%) | 10 (3%) | Reference |  |
| Grade 1 (6-7 points) | 353 (16%) | 24 (8%) | 3.28 (1.55-6.94) | 7 |
| Grade 2 (8-9 points) | 434 (20%) | 52 (18%) | 6.11 (3.06-12.19) | 3 |
| Grade 3 (≥10 points) | 898 (42%) | 213 (71%) | 13.96 (7.32-26.61) | 4 |

4) I would suggest to include more statistical measures of the predictive capacity of the model. Is it possible to calculate diagnostic accuracy, sensitivity, specificity, PPV, NPV and/or diagnostic likelihood ratios?

Response 4: Thank you for the suggestion. Sensitivity, specificity, PPV, NPV and Accuracy were added to Table 2 (please see Response 1 for these details)

5) It would be important that authors discuss about the differences between the models for nulliparous and multiparous and their implications. From their estimated coefficients, it seems like they only differ in the role of hypertensive disorders of pregnancy and (obviously) previous vaginal delivery.

Response 5: We agree with the reviewer that pregnancy-induced hypertension was significant among nulliparous women and not multi-parous women (S3 table). The following has been added to the discussion. (Line 261-264)

“Hypertensive disorders of pregnancy increased the risk of CS delivery (5). Nulliparous women have significant higher risk of developing hypertensive-related disorders compared to multiparous women (6,7). Similarly, we found pregnancy-induced hypertension increased the risk of emergency CS among nulliparous but not multiparous pregnant women.”

6) The model is of limited utility for settings where there is no information on CES-D. Since most likely this would be the case for most situations, I suggest to run separate models without this variable.

Response 6: The prediction model without CESD-score variable was included as S4 Table. The results from the prediction model without CESD-score (S4 Table) is comparable with the model with the CESD-score (Table 2). We also tested with CESD-score cut-off value of 16 which is associated with clinical depression and found the similar results.

**S4 Table.** Multiple logistic regression results excluding the CESD-score: (Training and validation dataset)

|  |  | Emergency CS  (Training, n=2150) | | | | Emergency CS  (Validation, n=530) | | | | | |
| --- | --- | --- | --- | --- | --- | --- | --- | --- | --- | --- | --- |
|  | Coefficient | | | Odds Ratio | 95% CI | | Coefficient | | Odds Ratio | 95% CI |  |
| Centered Age (years) | | | **0.05** | **1.05** | **1.02-1.08** | | **0.13** | | **1.14** | **1.07-1.22** |  |
| Centered Height (cm) | | | **-0.06** | **0.94** | **0.92-0.96** | | **-0.06** | | **0.94** | **0.91-0.98** |  |
| BMI in kg/m^2^ | | | **0.04** | **1.04** | **1.02-1.07** | | **0.08** | | **1.08** | **1.04-1.13** |  |
| CESD-score (ref: <10) | | | **-** | **-** | **-** | | **-** | | **-** | **-** |  |
| Hospital CS rate (CHILD) | | | 0.04 | 1.04 | 0.99-1.09 | | **0.16** | | **1.17** | **1.07-1.29** |  |
| Hypertensive Disorders of Pregnancy | | | **0.56** | **1.76** | **1.00-3.09** | | 0.16 | | 1.17 | 0.32-4.27 |  |
| Previous vaginal delivery | | | **-1.56** | **0.21** | **0.15-0.30** | | **-1.63** | | **0.20** | **0.10-0.38** |  |
| Constant | | | **-2.78** |  |  | | **-4.39** | |  |  |  |
| AUC | | |  | 0.74 | 0.71-0.77 | |  | | 0.78 | 0.73-0.83 |  |
| Sensitivity | | |  | 12% |  | | |  | 12% |  |  |
| Specificity | | |  | 99% |  | | |  | 99% |  |  |
| Positive Predictive Value | | |  | 38% |  | | |  | 69% |  |  |
| Negative Predictive Value | | |  | 87% |  | | |  | 86% |  |  |
| Accuracy | | |  | 87% |  | | |  | 85% |  |  |

**P-values <0.05 in bold;** AUC= area under curve; OR= odds ratio; CI= confidence interval;

7) How these results compare to other prediction model? I would suggest to comment about the WHO predictions models, such as C-Model by Souza et al.

Response 7: Thank you for the suggestion. The discussion was added accordingly (Line 254-258)

“The CS prediction models from Souza et al, a multicenter study, included maternal and fetal antenatal variables in addition to intrapartum factors such as cervical position, fetal station, fetal distress and fetal head molding (8). The AUC of our prediction model containing only antennal factors (0.74 training, 0.77 validation) was comparable to the model developed by Souza et al. (AUC 0.78).”

Reviewers' comments:

Reviewer's Responses to Questions

**Comments to the Author**

1. Is the manuscript technically sound, and do the data support the conclusions?

The manuscript must describe a technically sound piece of scientific research with data that supports the conclusions. Experiments must have been conducted rigorously, with appropriate controls, replication, and sample sizes. The conclusions must be drawn appropriately based on the data presented.

Reviewer #1: Partly

Reviewer #2: Partly

2. Has the statistical analysis been performed appropriately and rigorously?

Reviewer #1: Yes

Reviewer #2: Yes

3. Have the authors made all data underlying the findings in their manuscript fully available?

The [PLOS Data policy](http://www.plosone.org/static/policies.action#sharing) requires authors to make all data underlying the findings described in their manuscript fully available without restriction, with rare exception (please refer to the Data Availability Statement in the manuscript PDF file). The data should be provided as part of the manuscript or its supporting information, or deposited to a public repository. For example, in addition to summary statistics, the data points behind means, medians and variance measures should be available. If there are restrictions on publicly sharing data—e.g. participant privacy or use of data from a third party—those must be specified.

Reviewer #1: Yes

Reviewer #2: Yes

4. Is the manuscript presented in an intelligible fashion and written in standard English?

PLOS ONE does not copyedit accepted manuscripts, so the language in submitted articles must be clear, correct, and unambiguous. Any typographical or grammatical errors should be corrected at revision, so please note any specific errors here.

Reviewer #1: Yes

Reviewer #2: No

5. Review Comments to the Author

**Reviewer #1:**

1)The manuscript is relevant and reaches conclusions according to the objectives, but there is a lack of attemption to the analgesic modalities during labour and about the epidural rates. I think it is a very important issue when you are affording CS rates. Statistical analysis has been correctly performed and statistical material has been provided properly. The manuscript is written in correct english, as far as I know (I'm not native english speaker).
The authors should include details about analgesia during labour, epidural rate, and motor block, or at least to detail the epidural analgesic protocol that has been used in this cohort of women.

Response 1: Thank you for the suggestion. The purpose of this study is to predict the risk of emergency CS utilizing antenatal variables. Intrapartum variables including analgesia during labour (spinal, epidural, general anesthesia) were explicitly excluded from the prediction models. We have updated Table 1 to include the percentage of analgesia use (epidural, spinal and general anesthesia).

| Characteristics | Vaginal (n= 2,315) | Emergency CS (n=365) | Scheduled CS  (n=156) |
| --- | --- | --- | --- |
| Analgesia |  |  |  |
| Epidural | 1414 (61%) | 275 (75%) | 10 (6%) |
| Spinal | 27 (1%) | 90 (25%) | 151 (97%) |
| General Anesthesia | 5 (0.2%) | 23 (6.3%) | 5 (3%) |

**Reviewer #2:** This is an interesting study.

However,
**1) Some grammatical etc. errors should be corrected. Examples:**
Line 110/Please change “earlypregnancy” to “early pregnancy”

Line 119/Please change “cephalopevlic” to cephalopelvic” (using the advantage of automatic corrections).

Response 1: Thank you for pointing out the errors. They were corrected accordingly.

**2) In the “conclusion”, a real conclusion is expected (and not a detailed iteration of the title).**

Response 2: Thank you for the suggestion. The conclusion section was updated (line 333-341).

“ We developed a model to predict the likelihood of emergency CS using prenatal obstetric and non-obstetric factors. The proposed prediction model has similar performance characteristics compared to other emergency CS prediction models without the need for intra-partum prediction factors. The tool could be used in conjunction with the Grobman VBAC (Grobman et al., 2009) tool to assist in delivery mode decision-making and healthcare resource planning. Early identification of the women at an increased risk of emergency CS is important for patient management including referral for mental health counseling and weight management program to prevent emergency CS. Further prospective validation studies in the general population should be undertaken to confirm efficacy of the developed prediction model and the scoring system before application in the general population.”

**3) To take advantage of the practical benefits of similar studies, it should be clarified**:
a) the indications of emergency CS (ECS)

Response 3a: Thank you for the suggestion. Added in Line 65-69.

“Emergency CS is indicated when acute obstetrical complications that threaten the life of the mother and/ or the fetus including fetal distress and antepartum hemorrhage develop. As such, labour dystocia and fetal distress are common indications for emergency CS (5).

b) the necessary steps to avoid situations leading to ECS

Response 3b: Thank you for the suggestion. We have added in Line 270-277.

“Our prediction tool specifically excluded intra-partum factors associated with emergency CS such as labour dystocia and fetal distress. The contribution of labour dystocia and fetal distress on emergency CS rates are increasing area of focus for intervention. The American College of Obstetricians and Gynecologists (ACOG) guidelines for reducing the primary CS due to labour dystocia (5) were developed in 2014. As such, these guidelines were not implemented in the Society of Obstetricians and Gynaecologists of Canada (SOGC) guidelines (33) during the study recruitment period (2009-2012) (5). Further work will examine the role of our tool in the context SOGC guidelines for management of labour dystocia and fetal distress.

The antenatal obstetric and non-obstetric factors identified from our prediction tool can be utilized in screening and identification of individuals at high risk for an emergency CS. Increase surveillance and antennal interventions could be provided for the modifiable antenatal risk factors such as low dose aspirin for hypertension, counseling for depression and weight management for overweight pregnant women.”

**4) A working calculator could convince that the whole effort is a useful tool to justify or avoid ECS.**

Response: We agree with the reviewer that a publicly available questionnaire is important for knowledge translation. We are in the process of developing a web-based questionnaire to be hosted on our local research institutes website (<https://www.wchri.org/our-impact/patient-resources-created-by-our-members/>).

6. PLOS authors have the option to publish the peer review history of their article ([what does this mean?](https://journals.plos.org/plosone/s/editorial-and-peer-review-process#loc-peer-review-history)). If published, this will include your full peer review and any attached files.


If you choose “no”, your identity will remain anonymous but your review may still be made public.


**Do you want your identity to be public for this peer review?** For information about this choice, including consent withdrawal, please see our [Privacy Policy](https://www.plos.org/privacy-policy).

Reviewer #1: No

Reviewer #2: No
