# Supplementary material for: Prediction of odds for emergency cesarean section: A secondary analysis of the CHILD term birth cohort study
Source: PLoS One. 2022 Oct 6;17(10):e0268229. doi: 10.1371/journal.pone.0268229 (PMC9536615; doi:10.1371/journal.pone.0268229)
Supplement: Attachment — Submitted filename: Rebuttal.docx [file pone.0268229.s003.docx]

Review Comments to the Author

Reviewer #3: Overall, this study was interesting as they had a large number of samples and a combination of obstetrics and non-obstetrics factors counted for emergency cs prediction tools. However, there are a few things that could be added to improve the quality of the study.

In general, it was suggested to use appropriate written English language. Many paragraphs were considered not effective as repetitive words and redundant sentences were found.

- Abstract:
Keywords: antenatal depression?
Higher AUC in validation set?
Conclusion: Maybe you could paraphrase?

Response: Thank you for the suggestion. We have updated the abstract accordingly. Antenatal depression is synonymous with perinatal depression.

- Introduction
No reference 1? The reference started with number 2

Response: Updated the reference.

Line 64-66, 66-68: please paraphrase

Response: Rephrased the sentences (line 60-67)

Indications for scheduled CS can be divided into absolute and relative indications (1). Absolute indications for scheduled CS include cephalopelvic disproportion, placenta previa, abnormal lie and presentation. Prior CS delivery is a relative indication for scheduled CS ((1–5) .

Emergency CS is indicated when acute obstetrical complications that threaten the life of the mother and/ or the fetus including fetal distress and antepartum hemorrhage develop. Intrapartum factors such as labour dystocia, fetal distress, and umbilical cord prolapse are absolute indications for emergency CS (1,6,7)

- Methods & Results
When dividing the data? After recruitment or after applying exclusion criteria?

Response: The data were divided after applying the exclusion criteria.

Please specify with number too. The division of training and validation set (80% and 20%) was based on what?

Response: The division of training and validation data set (80% vs. 20%) was based on random splitting. Updated accordingly (line 131-134).

“Data analysis steps to develop an emergency CS score are described in **Figure 1**. A total of 2,836 pregnant women met the inclusion criteria. First, the data were randomly divided into two groups: a training dataset (80% of the sample, n=2,269) and a validation dataset (20% of the sample, n=567).”

The use of CESD in methods, please put a reference.

Response: Reference added (line 108).

Diagnosis criteria for hypertensive disorder and diabetes mellitus complicating pregnancy? please put reference too

Response: References added (line 106)

The Adjusted OR, please specify, adjusted with what?

Response: updated accordingly in the methods section (line 152-153)

“The final model was adjusted for maternal height, BMI, CESD-score, hypertensive disorders of pregnancy, history of previous vaginal delivery and hospital CS rate.”

- Analysis:
This study focuses in comparing vaginal birth and emergency cs. Since you put scheduled cs in the table 1, why don’t you also compare the scheduled cs and the emergency cs? The findings might be interesting and can be added in the discussion too.

Response: Thank you for the suggestion. The main purpose of this study is to develop an emergency CS risk prediction tool. The study excluded major indications for scheduled CS (i.e. cephalopelvic disproportion, placenta previa, previous CS delivery) in the analysis. Additionally, several tools for predicting CS already exist.

- Discussion:
Please add discussion to compare other studies for emergency cs scoring systems. Not only study for cs prediction in general. Such as a current previous study which develop a scoring system for emergency cs by maternal-fetal perinatal characteristics. Although that one was different from yours, maybe you can add this in the discussion.

Response: We updated with the emergency CS scoring system (line 253-254).
“The emergency CS risk scoring system by Guan et al (2020) (3), with an AUC of 0.79, included intrapartum factors such as quantity and color of the amniotic fluid.”

Typo: Line 144, 155, 249

Response: Typo errors corrected (lines 145, 158, 252)

- Reference:
A number of references were considered too old, there are many newer references that could be used related to the issue

Response: Updated the references

- Supplementary materials:
Table 1: acupressure? Reference?

Response: reference updated

Table 2 in supplementary files similar to Table 1 in the manuscript? No need to mention it in supplementary files.

Response: Table 1 in the manuscript shows the demographic, antenatal and obstetric characteristics associated with mode of delivery. Table 2 in supplementary files presents the demographic, antenatal and obstetric characteristics of Training and Validation data (line 134-135)

“The demographic, antenatal and obstetric characteristics of training and validation data set was shown in **S2 Table**. “

Reviewer #4: The authors have done a good job attending the reviews, especially the one related to the comparison with other tools predicting the risk of C-Section. However, I think it is still possible in the discussion to stress what are the advantages of this tool. The authors must make a strong case for why this new tool is necessary, and the advantages of using this one instead of other existing tools up front.

Response: We have updated accordingly. (line 274-277)

“The components of our eCS score including maternal age, height, BMI, childbirth history and hypertensive disorders of pregnancy are often collected as part of routine prenatal care. Additionally, unlike prior emergency CS tools, our score does not include any intrapartum data allowing for application at any point during pregnancy.”
