# Supplementary material for: Prediction of odds for emergency cesarean section: A secondary analysis of the CHILD term birth cohort study
Source: PLoS One. 2022 Oct 6;17(10):e0268229. doi: 10.1371/journal.pone.0268229 (PMC9536615; doi:10.1371/journal.pone.0268229)
Supplement: Attachment — Submitted filename: 2022-04-20 PLOS one R3 response.docx [file pone.0268229.s004.docx]

Thank you for the insightful comments and the opportunity to further improve our manuscript. We have provided responses to each of the editor’s comments and have amended the manuscript accordingly.

**Query 1:** I would request authors to comment on the implications of their tool's low sensitivity in the discussion. Does that mean the tool works better as a "rule-in" rather than a "rule-out" test? And if so, do the relatively high AUC and accuracy result not from identifying (predicting) those who need an emergency CS but those who would not need one?

*Response:* We agree that additional clarification of the tool will help the readers understand the tool’s applicability in practice. The high specificity and low sensitivity suggest that the tool is better at determining who will **not** need an eCS (a rule-out test). The editor is correct that the high AUC and accuracy are from identifying those who do not need an eCS (the majority of women). We would recommend that women who screen positive have closer pre-natal follow-up rather than scheduling a CS. The tool also identifies areas for potential intervention to lower an individuals risk for an eCS. We have added the following to the discussion (Lines 305 – 311)

“The high specificity and low sensitivity suggest that the tool is good at determining who will not need an eCS (a rule-out test). As such, we would recommend that women who screen positive have closer pre-natal follow-up. The tool identifies areas for potential intervention to lower an individual’s risk for an eCS.”

**Query 2:** Please also confirm that PPV in the validation sample is 63% (Table 2).

*Response:* We have confirmed that the PPV of the validation sample is 63%. We have attached the classification table for the validation dataset in this response (appendix 1).

Appendix 1: 2x2 classification table for validation datasets among those with complete data for all predictor variables.
